# Omicron infection enhances Delta antibody immunity in vaccinated persons

**DOI:** 10.1038/s41586-022-04830-x

**Published:** 2022-05-06

**Authors:** Khadija Khan, Farina Karim, Sandile Cele, Kajal Reedoy, James Emmanuel San, Gila Lustig, Houriiyah Tegally, Yuval Rosenberg, Mallory Bernstein, Zesuliwe Jule, Yashica Ganga, Nokuthula Ngcobo, Matilda Mazibuko, Ntombifuthi Mthabela, Zoey Mhlane, Nikiwe Mbatha, Yoliswa Miya, Jennifer Giandhari, Yajna Ramphal, Taryn Naidoo, Aida Sivro, Natasha Samsunder, Ayesha B. M. Kharsany, Daniel Amoako, Jinal N. Bhiman, Nithendra Manickchund, Quarraisha Abdool Karim, Nombulelo Magula, Salim S. Abdool Karim, Glenda Gray, Willem Hanekom, Anne von Gottberg, COMMIT-KZN Team, Rohen Harrichandparsad, Kobus Herbst, Prakash Jeena, Thandeka Khoza, Henrik Kløverpris, Alasdair Leslie, Rajhmun Madansein, Mohlopheni Marakalala, Mosa Moshabela, Kogie Naidoo, Zaza Ndhlovu, Thumbi Ndung’u, Kennedy Nyamande, Vinod Patel, Theresa Smit, Adrie Steyn, Emily Wong, Ron Milo, Bernadett I. Gosnell, Richard J. Lessells, Penny L. Moore, Tulio de Oliveira, Mahomed-Yunus S. Moosa, Alex Sigal

**Affiliations:** 1 Africa Health Research Institute, Durban, South Africa; 2 School of Laboratory Medicine and Medical Sciences, University of KwaZulu-Natal, Durban, South Africa; 3 KwaZulu-Natal Research Innovation and Sequencing Platform, Durban, South Africa; 4 Centre for the AIDS Programme of Research in South Africa, Durban, South Africa; 5 Centre for Epidemic Response and Innovation, School of Data Science and Computational Thinking, Stellenbosch University, Stellenbosch, South Africa; 6 Department of Plant and Environmental Sciences, Weizmann Institute of Science, Rehovot, Israel; 7 Department of Medical Microbiology, University of KwaZulu-Natal, Durban, South Africa; 8 National Institute for Communicable Diseases of the National Health Laboratory Service, Johannesburg, South Africa; 9 Department of Infectious Diseases, Nelson R. Mandela School of Clinical Medicine, University of KwaZulu-Natal, Durban, South Africa; 10 Department of Epidemiology, Mailman School of Public Health, Columbia University, New York, NY USA; 11 Department of Internal Medicine, Nelson R. Mandela School of Medicine, University of Kwa-Zulu Natal, Durban, South Africa; 12 South African Medical Research Council, Cape Town, South Africa; 13 Division of Infection and Immunity, University College London, London, UK; 14 School of Pathology, Faculty of Health Sciences, University of Witwatersrand, Johannesburg, South Africa; 15 SAMRC Antibody Immunity Research Unit, School of Pathology, Faculty of Health Sciences, University of the Witwatersrand, Johannesburg, South Africa; 16 Institute of Infectious Disease and Molecular Medicine, University of Cape Town, Cape Town, South Africa; 17 Department of Global Health, University of Washington, Seattle, WA USA; 18 Max Planck Institute for Infection Biology, Berlin, Germany; 19 Department of Neurosurgery, University of KwaZulu-Natal, Durban, South Africa; 20 South African Population Research Infrastructure Network, Durban, South Africa; 21 Department of Paediatrics and Child Health, University of KwaZulu-Natal, Durban, South Africa; 22 Department of Immunology and Microbiology, University of Copenhagen, Copenhagen, Denmark; 23 Department of Cardiothoracic Surgery, University of KwaZulu-Natal, Durban, South Africa; 24 College of Health Sciences, University of KwaZulu-Natal, Durban, South Africa; 25 Ragon Institute of MGH, MIT and Harvard, Boston, MA USA; 26 HIV Pathogenesis Programme, The Doris Duke Medical Research Institute, University of KwaZulu-Natal, Durban, South Africa; 27 Department of Pulmonology and Critical Care, University of KwaZulu-Natal, Durban, South Africa; 28 Department of Neurology, University of KwaZulu-Natal, Durban, South Africa; 29 Division of Infectious Diseases, University of Alabama at Birmingham, Birmingham, AL USA

**Keywords:** SARS-CoV-2, Viral infection

## Abstract

The extent to which Omicron infection^1–9^, with or without previous vaccination, elicits protection against the previously dominant Delta (B.1.617.2) variant is unclear. Here we measured the neutralization capacity against variants of severe acute respiratory syndrome coronavirus 2 in 39 individuals in South Africa infected with the Omicron sublineage BA.1 starting at a median of 6 (interquartile range 3–9) days post symptom onset and continuing until last follow-up sample available, a median of 23 (interquartile range 19–27) days post symptoms to allow BA.1-elicited neutralizing immunity time to develop. Fifteen participants were vaccinated with Pfizer's BNT162b2 or Johnson & Johnson's Ad26.CoV2.S and had BA.1 breakthrough infections, and 24 were unvaccinated. BA.1 neutralization increased from a geometric mean 50% focus reduction neutralization test titre of 42 at enrolment to 575 at the last follow-up time point (13.6-fold) in vaccinated participants and from 46 to 272 (6.0-fold) in unvaccinated participants. Delta virus neutralization also increased, from 192 to 1,091 (5.7-fold) in vaccinated participants and from 28 to 91 (3.0-fold) in unvaccinated participants. At the last time point, unvaccinated individuals infected with BA.1 had low absolute levels of neutralization for the non-BA.1 viruses and 2.2-fold lower BA.1 neutralization, 12.0-fold lower Delta neutralization, 9.6-fold lower Beta variant neutralization, 17.9-fold lower ancestral virus neutralization and 4.8-fold lower Omicron sublineage BA.2 neutralization relative to vaccinated individuals infected with BA.1. These results indicate that hybrid immunity formed by vaccination and Omicron BA.1 infection should be protective against Delta and other variants. By contrast, infection with Omicron BA.1 alone offers limited cross-protection despite moderate enhancement.

## Abstract (web summary)

A study quantifying the neutralization of severe acute respiratory syndrome coronavirus 2 variants in individuals infected with Omicron/BA.1 shows that vaccinated individuals previously infected with Omicron have enhanced protection against reinfection with current variants, \including Omicron/BA.2, while Omicron/BA.1 infected unvaccinated individuals have limited protection.

## Main

The Omicron variant of severe acute respiratory syndrome coronavirus 2 (SARS-CoV-2), first identified in November 2021 in South Africa and Botswana^10^, has been shown by us^1^ and others^2–9^ to have extensive but incomplete escape from neutralizing immunity elicited by vaccines and previous infection, with boosted individuals showing better neutralization. In South Africa, Omicron infections led to a lower incidence of severe disease relative to other variants^11,12^, although this can be at least partly explained by pre-existing immunity^13^. The first Omicron sublineage to appear was BA.1, which was supplanted by the BA.2 sublineage in many countries^14^.

How Omicron BA.1 infection will interact with vaccination to protect against the previously dominant Delta variant, emerging variants such as BA.2 and other variants is still unclear. We isolated live Omicron BA.1, Omicron BA.2, ancestral, Beta and Delta viruses and neutralized viruses with plasma from participants enrolled and longitudinally sampled during the Omicron BA.1 infection wave in South Africa, with all participants having a confirmed diagnosis of SARS-CoV-2 by quantitative PCR. To quantify neutralization, we used a live virus neutralization assay and calculated the focus reduction neutralization test (FRNT_50_) titre, the inverse of the plasma dilution required for 50% neutralization, as measured by the reduction in the number of infection foci. We enrolled 41 participants who reported symptoms from late November 2021 to January 2022. We successfully sequenced the infecting virus in 26 participants, and all sequences corresponded to Omicron BA.1 (Extended Data Table 1). Two participants had advanced human immunodeficiency virus (HIV) disease on the basis of a low CD4 count (<200 cells μl^−1^ throughout the study) and unsuppressed HIV infection, and we excluded these participants because of our previous data showing an atypical response to SARS-CoV-2 in advanced HIV disease^15^. Extended Data Table 2 summarizes the characteristics of the remaining 39 participants.

Of the 39 participants, 27 were admitted to hospital because of coronavirus disease 2019 symptoms. Seven required supplemental oxygen and one died. Fifteen participants were vaccinated and had a breakthrough Omicron BA.1 infection. The median time post vaccination was 139 days (interquartile range (IQR) 120–178), a time interval that would predict considerable waning of the vaccine-elicited immune response^16^, which may have contributed to the breakthrough infections. Eight participants were vaccinated with two doses of Pfizer's BNT162b2 and seven were vaccinated with Johnson & Johnson's Ad26.CoV2.S (six with a single dose and one with two doses; Extended Data Table 1). The length of hospital stay was shorter in the vaccinated (3.5 days) relative to unvaccinated (8 days; Extended Data Table 2) participants. Three participants self-reported having a previous SARS-CoV-2 infection (Extended Data Table 1).

**Extended Data Fig. 1:**
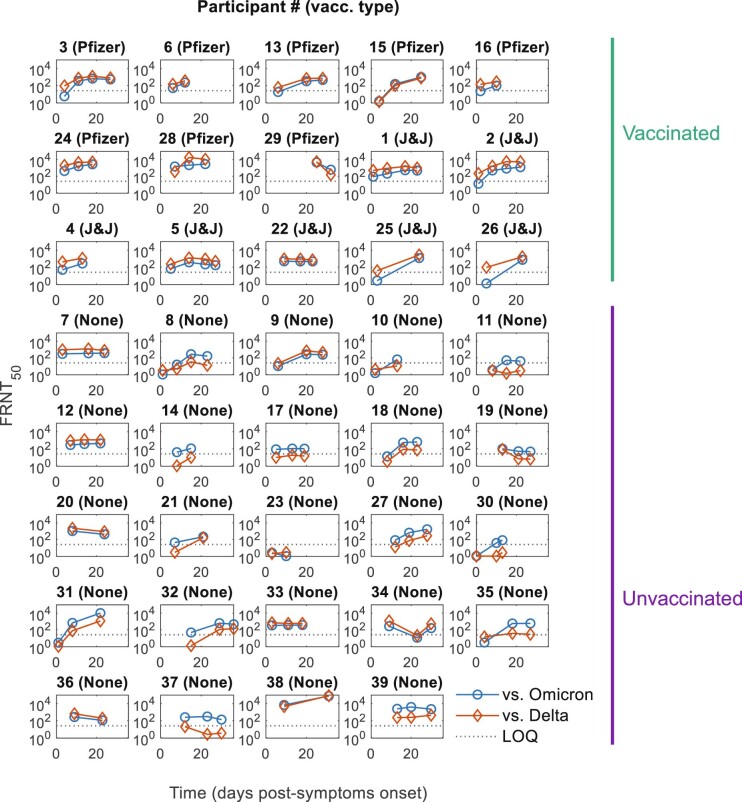
Longitudinal Omicron/BA.1 and Delta neutralization capacity in Omicron/BA.1 infected participants. Neutralization of Omicron (blue) and Delta (red) at all study visits. Participant number is as in Extended Data Table 1. Top three rows are participants vaccinated with Pfizer BNT162b2 (n = 8) or Johnson and Johnson Ad26.CoV2.S (n = 7) and bottom five rows are unvaccinated participants (n = 24). X-axis is the time post-symptom onset when sample was collected, and y-axis is neutralization as FRNT_50_. Dashed line is the most concentrated plasma tested (LOQ, limit of quantification below which FRNT_50_ values are extrapolated). All participants recovered except participant 29, who died.

Participants were sampled at enrolment at a median of 6 days (IQR 3–9 days) after symptom onset, and again at weekly follow-up visits that were attended as practicable because of the Christmas holidays in South Africa. The last follow-up visit was a median of 23 days (IQR 19–27 days) post-symptom onset (Extended Data Table 1). Examining neutralization at all available time points per study participant showed that neutralization of the Omicron BA.1 variant increased substantially in most participants from enrolment to the time of the last follow-up (Extended Data Fig. 1), consistent with developing a neutralizing antibody response to Omicron BA.1 infection. We therefore analysed neutralization at enrolment (baseline for the study) and the last follow-up visit to quantify the increase in neutralization capacity after Omicron infection.

**Fig. 1:**
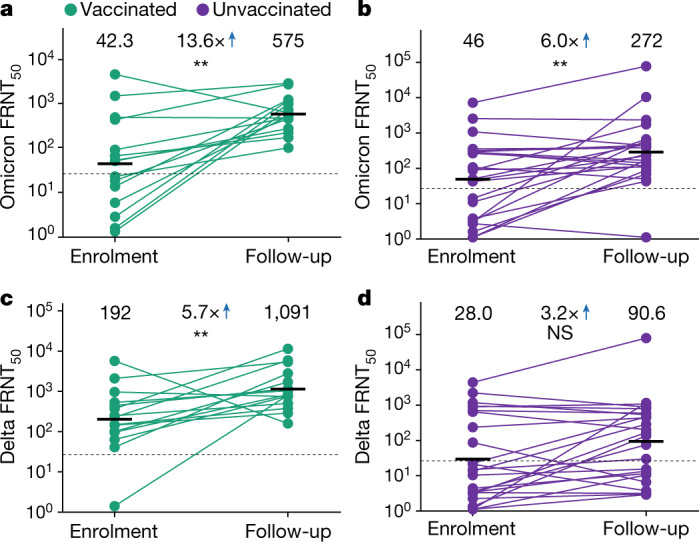
Enhancement of Delta neutralization by Omicron infection. **a**,**b**, Neutralization of the Omicron BA.1 virus over time for *n* = 15 vaccinated (**a**) and *n* = 24 unvaccinated (**b**) participants infected with Omicron BA.1. **c**,**d**, Neutralization of the Delta virus over time for the same vaccinated (*n* = 15; **c**) and unvaccinated (*n* = 24; **d**) participants as in **a**,**b**. For each participant, the sample collected at the initial enrolment visit (median 6 days post symptom onset) was compared with that collected at the last follow-up visit (median 23 days post symptom onset). The neutralization capacity per participant was determined in two independent experiments, and the numbers and horizontal bars are GMTs over all participants per group of the reciprocal plasma dilution (FRNT_50_) resulting in 50% neutralization. The fold change was calculated by dividing the GMT from the follow-up by the GMT from the enrolment visit. The dashed line is the most concentrated plasma tested. The *P* values were determined by a left-sided Wilcoxon rank sum test measuring the significance of the increase; **P = 0.01–0.001; NS, not significant. The exact *P* values are 0.0012 (**a**), 0.0081 (**b**), 0.0021 (**c**) and 0.11 (**d**). Source data

We observed that Omicron BA.1 neutralization increased in vaccinated individuals from a low geometric mean titre (GMT) FRNT_50_ of 42 at the enrolment visit to 575 at the last follow-up visit about 2 to 3 weeks later, a 13.6-fold change (95% CI confidence interval (CI) 3.7–50.2; Fig. 1a). The samples from unvaccinated participants at the study baseline neutralized Omicron BA.1 at a similar starting level of 46 and reached a final level of 272 at the last follow-up, a 6.0-fold increase (95% CI 2.2–16.1; Fig. 1b). Neutralization of the Delta virus also increased during this period. At enrolment, neutralization capacity against the Delta virus was 192 and reached a final level of 1,091 at the last follow-up visit in vaccinated participants, a 5.7-fold increase (95% CI 1.7–18.4; Fig. 1c). Unvaccinated participants had lower Delta neutralization at baseline with Delta virus FRNT_50_ = 28, and reached FRNT_50_ = 91, a 3.2-fold increase (95% CI 1.3–8.1; Fig. 1d).

**Fig. 2:**
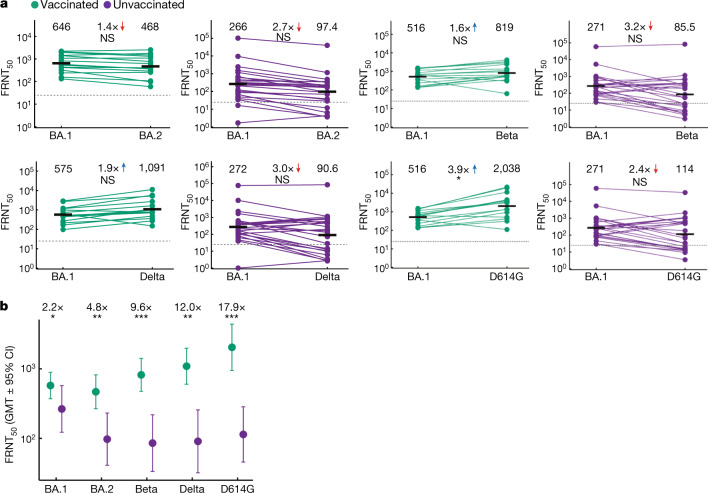
Gap in neutralizing immunity between vaccinated and unvaccinated participants infected with Omicron BA.1. **a**, Neutralization of Omicron BA.2, Beta, Delta and ancestral (with the D614G substitution) viruses compared to the Omicron BA.1 virus at the last available follow-up time point in *n* = 15 vaccinated or *n* = 24 unvaccinated participants infected with Omicron BA.1. The neutralization capacity per participant was determined in two independent experiments, and the numbers and horizontal bars are GMT FRNT_50_. The fold change was calculated by dividing the larger by the smaller GMT. The dashed line is the most concentrated plasma tested. The *P* values were determined by a two-sided Wilcoxon rank sum test; *P = 0.05–0.01; NS, not significant. The exact *P* values (vaccinated/unvaccinated) are: 0.22/0.087 for BA.2, 0.36/0.071 for Beta, 0.15/0.25 for Delta and 0.014/0.20 for ancestral. **b**, Comparison of the neutralization capacity against the Omicron BA.1, Omicron BA.2, Beta, Delta and ancestral (D614G) viruses in vaccinated (*n* = 15) versus unvaccinated (*n* = 24) participants infected with Omicron BA.1. The neutralization capacity per participant was determined in two independent experiments for all strains except for Omicron BA.1, for which six experiments were available and were used in the calculation. The points represent GMT FRNT_50_ per group and the error bars are GMT 95% CIs. The *P* values were determined by a two-sided Wilcoxon rank sum test; *P = 0.05–0.01; **P = 0.01–0.001; ***P = 0.001–0.0001. The exact *P* values are 0.025 (BA.1), 0.0026 (BA.2), 4.1 × 10^−4^ (Beta), 0.0012 (Delta) and 3.3 × 10^−4^ (ancestral). Source data

**Extended Data Fig. 2:**
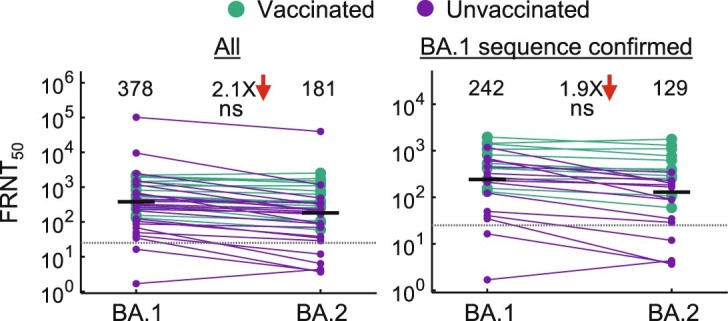
Fold-drop in BA.2 versus BA.1 neutralization in all and sequence confirmed samples. Neutralization of Omicron BA.2 compared to BA.1 in participants described in Extended Data Table 1, excluding participant 14 for technical reasons and participants 40 and 41 because of advanced HIV disease. Left panel shows neutralization capacity in all n = 38 participants and right panel shows neutralization capacity for n = 25 participants where infection was successfully sequenced and determined to be BA.1. Dashed line is the most concentrated plasma tested. p-values were 0.077 for all and 0.15 for BA.1 sequence confirmed participants as determined by a two-sided Wilcoxon rank sum test. ns, not significant.

We next compared Omicron BA.1 to Omicron BA.2, Delta, Beta (ref. ^17^) and ancestral virus neutralization at the last available follow-up visit in three sets of paired experiments, each comparing Omicron BA.2, Delta or ancestral and Beta virus neutralization to Omicron BA.1 neutralization. The range of Omicron BA.1 neutralization shown in Fig. 2a for different experiments (FRNT_50_ = 516 to 646 for vaccinated samples and 266 to 271 for unvaccinated samples) is the result of experimental variation. BA.2 neutralization was moderately and not significantly lower relative to BA.1 neutralization in both vaccinated and unvaccinated participants. Testing only participants with sequence-confirmed Omicron BA.1 infection gave a similar result (Extended Data Fig. 2). The trend for the other variants and the ancestral virus was that neutralization was higher relative to Omicron BA.1 in vaccinated participants but lower relative to Omicron BA.1 in unvaccinated participants, although the differences were mostly not significant (Fig. 2a). As a result of the relatively moderate fold change, higher participant numbers would probably be required to make the trends statistically significant.

The comparison of the other variants to Omicron BA.1 within the vaccinated or unvaccinated group does not indicate the differences in neutralization capacity elicited by Omicron BA.1 between the vaccinated and unvaccinated participants. We therefore compared neutralization of each variant between the vaccinated and unvaccinated groups at the last time point directly (Fig. 2b). The smallest difference between vaccinated and unvaccinated participants was in neutralization of Omicron BA.1, the infecting variant, with the vaccinated participants showing 2.2-fold higher neutralization. For the other variants, neutralization was higher in vaccinated participants by a factor of 4.8-fold for Omicron BA.2, 9.6-fold for Beta, 12.0-fold for Delta and 17.9-fold for ancestral (Fig. 2b). All differences were significant, and the 95% CIs for the GMT FRNT_50_ of vaccinated and unvaccinated participants did not overlap for BA.2, Beta, Delta or ancestral virus neutralization (Fig. 2b). For the unvaccinated participants, the absolute neutralization capacity against the BA.2, Beta, Delta and ancestral viruses was low^18^, with GMT FRNT_50_ being about or below FRNT_50_ = 100 (Fig. 2b).

**Fig. 3:**
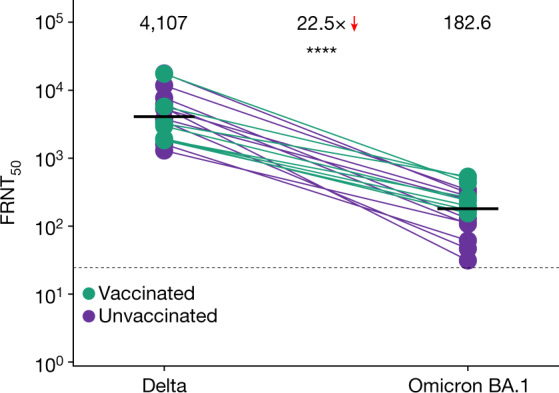
Escape of Omicron virus from Delta-infection-elicited immunity. Neutralization of Delta compared to the Omicron BA.1 virus by Delta-infection-elicited plasma immunity in vaccinated and unvaccinated participants. A total of 18 samples were tested from *n* = 14 participants infected during the Delta infection wave in South Africa. The neutralization capacity per participant was determined in two independent experiments, and the numbers and horizontal bars are GMT FRNT_50_. The fold change was calculated by dividing the larger by the smaller GMT. The dashed line is the most concentrated plasma tested. ****P = 3.2 × 10^−7^ as determined by a two-sided Wilcoxon rank sum test. Source data

We also tested neutralization of Omicron BA.1 by Delta-variant-elicited immunity. We collected 18 plasma samples from 14 participants (including pre-vaccination and post-vaccination samples from 4 participants) previously infected in the Delta variant wave in South Africa, 8 of whom were vaccinated either before or after infection (Extended Data Table 3). Confirming previously reported results^19^, we observed similar escape of Omicron BA.1 from Delta-elicited immunity across all samples tested, manifested as a 22.5-fold decrease (95% CI 14.4–35.0) in Omicron BA.1 neutralization compared to Delta virus neutralization (Fig. 3).

The large fold drop in Delta-infection-elicited neutralization capacity against Omicron BA.1 contrasts with the moderate and nonsignificant fold drops, or even fold increases, in neutralization of other variants relative to Omicron BA.1 in individuals infected with Omicron BA.1. However, in unvaccinated individuals, even though fold drops in neutralization were moderate and nonsignificant, the absolute levels of neutralization of the other variants, and of Omicron BA.1 itself, were low and on a similar scale to the cross-neutralization capacity against Omicron in Delta-infection-elicited immunity. This is consistent with other recently reported results^20^, and possibly indicates that Omicron is poorly immunogenic. In agreement with recent reports^21,22^, our observations show moderately and nonsignificantly lower neutralization of BA.2 by BA.1-elicited immunity. The results explain epidemiological observations showing that Omicron BA.2 reinfection is relatively rare soon after Omicron BA.1 infection^23,24^.

Our results may be supportive of a scenario in which hybrid immunity formed by Omicron infection combined with vaccination protects as well or better against reinfection with variants such as Delta relative to reinfection with Omicron itself. By contrast, unvaccinated participants infected with Omicron BA.1 only, have low neutralization capacity against the Omicron BA.2, Beta, Delta and ancestral viruses.

Limitations of this study include heterogeneity in participant immune history, including two vaccination types and one boost. On the basis of the high seroprevalence observed in South Africa^25,26^, some participants may also have had unreported previous infection. However, including two vaccine types did not mask the differences between vaccinated and unvaccinated participants, and the low levels of neutralization in unvaccinated participants against the ancestral, Beta and Delta viruses (the dominant strains in the preceding South African infection waves) support the notion that these participants were either not previously infected, or that immunity has waned completely. Participants were also mostly hospitalized, which may not be typical of Omicron infection^13,27^. Increased disease severity has been shown to lead to higher anti-SARS-CoV-2 antibody titres^28^. This should help in the detection of the neutralization response, but whether it would affect the trend we observed is unclear. Omicron infection is unlikely benign to the extent that hospitalization is an outlier outcome: in the USA, the number of individuals with coronavirus disease 2019 who died in the Omicron wave was similar to the number who died in the Delta wave^27^. Neutralizing immunity may have increased further in some participants had we sampled later: the neutralizing capacity did not plateau at the last time point in 8 of the 24 (33%) unvaccinated participants (participants 10, 14, 21, 27, 30, 31, 34 and 38; Extended Data Fig. 1) and 6 of the 15 (40%) vaccinated participants (participants 4, 6, 15, 16, 25 and 26). Therefore, the temporal dynamics give no clear indication that the immunity in the unvaccinated participants was delayed and would have reached similar levels to that of vaccinated participants if sampled later. However, the consequences of waning immunity several months post Omicron infection should be investigated.

The gap in immunity between unvaccinated individuals infected with Omicron BA.1 and vaccinated individuals with BA.1 breakthrough infection is concerning. Especially as immunity wanes, unvaccinated individuals post Omicron infection are likely to have poor cross-protection against existing and possibly emerging SARS-CoV-2 variants, despite acquiring some neutralizing immunity to the infecting Omicron BA.1 sub-lineage variant. The implication may be that Omicron BA.1 infection alone is not sufficient for protection and vaccination should be administered even in areas with a high prevalence of Omicron infection to protect against other variants.

## Methods

### Informed consent and ethics

Blood samples and the Delta isolate were obtained after written informed consent from adults with PCR-confirmed SARS-CoV-2 infection who were enrolled in a prospective cohort study at the Africa Health Research Institute approved by the Biomedical Research Ethics Committee at the University of KwaZulu-Natal (reference BREC/00001275/2020). Omicron BA.1 was isolated from a residual swab sample for SARS-CoV-2 where isolation from the sample was approved by the University of the Witwatersrand Human Research Ethics Committee (HREC; reference M210752). The sample to isolate Omicron BA.2 was collected after written informed consent as part of the study “COVID-19 transmission and natural history in KwaZulu-Natal, South Africa: Epidemiological Investigation to Guide Prevention and Clinical Care” of the Centre for the AIDS Programme of Research in South Africa (CAPRISA) and isolation from the sample approved by the Biomedical Research Ethics Committee at the University of KwaZulu-Natal (reference BREC/00001195/2020, BREC/00003106/2021).

### Reagent availability

Virus isolates and the cell line are available from the corresponding author. A Biosafety Level 3 facility is required for laboratories receiving live SARS-CoV-2.

### Whole-genome sequencing, genome assembly and phylogenetic analysis

RNA was extracted on an automated Chemagic 360 instrument, using the CMG-1049 kit (Perkin Elmer). The RNA was stored at −80 °C before use. Libraries for whole-genome sequencing were prepared using either the Oxford Nanopore Midnight protocol with Rapid Barcoding or the Illumina COVIDseq Assay. For the Illumina COVIDseq assay, the libraries were prepared according to the manufacturer’s protocol. Briefly, amplicons were tagmented, followed by indexing using the Nextera UD Indexes Set A. Sequencing libraries were pooled, normalized to 4 nM and denatured with 0.2 N sodium acetate. An 8 pM sample library was spiked with 1% PhiX (PhiX Control v3 adaptor-ligated library used as a control). We sequenced libraries on a 500-cycle v2 MiSeq Reagent Kit on the Illumina MiSeq instrument (Illumina). On the Illumina NextSeq 550 instrument, sequencing was performed using the Illumina COVIDSeq protocol (Illumina), an amplicon-based next-generation sequencing approach. The first-strand synthesis was carried using random hexamer primers from Illumina, and the synthesized cDNA underwent two separate multiplex PCR reactions. The pooled PCR-amplified products were processed for tagmentation and adaptor ligation using IDT for Illumina Nextera UD Indexes. Further enrichment and cleanup was performed as per protocols provided by the manufacturer (Illumina). Pooled samples were quantified using a Qubit 3.0 or 4.0 fluorometer (Invitrogen) through the Qubit dsDNA High Sensitivity Assay according to the manufacturer’s instructions. The fragment sizes were analysed using TapeStation 4200 (Invitrogen). The pooled libraries were further normalized to 4 nM concentration and 25 μl of each normalized pool containing unique index adaptor sets was combined in a new tube. The final library pool was denatured and neutralized with 0.2 N sodium hydroxide and 200 mM Tris-HCl (pH 7), respectively. A 1.5 pM sample library was spiked with 2% PhiX. Libraries were loaded onto a 300-cycle NextSeq 500/550 HighOutput Kit v2 and run on the Illumina NextSeq 550 instrument (Illumina). For Oxford Nanopore sequencing, the Midnight primer kit was used: cDNA synthesis was performed on the extracted RNA using LunaScript RT mastermix (New England BioLabs) followed by gene-specific multiplex PCR using the Midnight Primer pools that produce 1,200-base-pair amplicons that overlap to cover the 30-kb SARS-CoV-2 genome. Amplicons from each pool were pooled and used neat for barcoding with the Oxford Nanopore Rapid Barcoding kit as per the manufacturer’s protocol. Barcoded samples were pooled and bead purified. After the bead cleanup, the library was loaded on a prepared R9.4.1 flow cell. A GridION X5 or MinION sequencing run was initiated using MinKNOW software with the base-call setting switched off. We assembled paired-end and nanopore.fastq reads using Genome Detective 1.132 (https://www.genomedetective.com), which was updated for the accurate assembly and variant calling of tiled primer amplicon Illumina or Oxford Nanopore reads, and the Coronavirus Typing Tool. For Illumina assembly, the GATK HaploTypeCaller –min-pruning 0 argument was added to increase mutation calling sensitivity near sequencing gaps. For Nanopore, low-coverage regions with poor alignment quality (<85% variant homogeneity) near sequencing/amplicon ends were masked to be robust against primer drop-out experienced in the spike gene, and the sensitivity for detecting short inserts using a region-local global alignment of reads was increased. In addition, we also used the wf_artic (ARTIC SARS-CoV-2) pipeline as built using the nextflow workflow framework. In some instances, mutations were confirmed visually with .bam files using Geneious software V2020.1.2 (Biomatters). The reference genome used throughout the assembly process was NC_045512.2 (numbering equivalent to MN908947.3). For lineage classification, we used the widespread dynamic lineage classification method from the Phylogenetic Assignment of Named Global Outbreak Lineages software suite (https://github.com/hCoV-2019/pangolin).

### Cells

Vero E6 cells (originally ATCC CRL-1586, obtained from Cellonex in South Africa) were propagated in complete growth medium consisting of Dulbecco's modified Eagle medium with 10% fetal bovine serum (Hyclone) containing 10 mM HEPES, 1 mM sodium pyruvate, 2 mM l-glutamine and 0.1 mM nonessential amino acids (Sigma-Aldrich). Vero E6 cells were passaged every 3–4 days. The H1299-E3 cell line (H1299 originally from ATCC as CRL-5803) was propagated in growth medium consisting of complete Roswell Park Memorial Institute 1640 medium with 10% fetal bovine serum containing 10 mM HEPES, 1 mM sodium pyruvate, 2 mM l-glutamine and 0.1 mM nonessential amino acids. Cells were passaged every second day. The H1299-E3 (H1299-ACE2, clone E3) cell line was derived from H1299 as described in our previous work^1,17^. Cell lines were not authenticated. Cell lines tested negative for mycoplasma contamination.

### Virus expansion

All work with live virus was performed in Biosafety Level 3 containment using protocols for SARS-CoV-2 approved by the Africa Health Research Institute Biosafety Committee. ACE2-expressing H1299-E3 cells were seeded at 4.5 × 10^5^ cells in a 6-well plate well and incubated for 18–20 h. After one Dulbecco's phosphate-buffered saline (PBS) wash, the subconfluent cell monolayer was inoculated with 500 μl universal transport medium diluted 1:1 with growth medium filtered through a 0.45-μm filter. Cells were incubated for 1 h. Wells were then filled with 3 ml complete growth medium. After 4 days of infection (completion of passage 1 (P1)), cells were trypsinized, centrifuged at 300 RCF for 3 min and resuspended in 4 ml growth medium. All cells from the P1 infection were added to Vero E6 cells that had been seeded at 2 × 10^5^ cells ml^−1^, 20 ml total, 18–20 h earlier in a T75 flask for cell-to-cell infection. The coculture of ACE2-expressing H1299-E3 and Vero E6 cells was incubated for 1 h, and the flask was filled with 20 ml of complete growth medium and incubated for 4 days. The viral supernatant from this culture (passage 2 (P2) stock) was used for experiments.

### Live virus neutralization assay

H1299-E3 cells were plated in a 96-well plate (Corning) at 30,000 cells per well 1 day pre-infection. Plasma was separated from EDTA-anticoagulated blood by centrifugation at 500 RCF for 10 min and stored at −80 °C. Aliquots of plasma samples were heat-inactivated at 56 °C for 30 min and clarified by centrifugation at 10,000 RCF for 5 min. Virus stocks were used at approximately 50–100 focus-forming units per microwell and added to diluted plasma. Antibody–virus mixtures were incubated for 1 h at 37 °C, 5% CO_2_. Cells were infected with 100 μl of the virus–antibody mixtures for 1 h, and then 100 μl of a 1× Roswell Park Memorial Institute 1640 (Sigma-Aldrich, R6504), 1.5% carboxymethylcellulose (Sigma-Aldrich, C4888) overlay was added without removing the inoculum. Cells were fixed 18 h after infection using 4% paraformaldehyde (Sigma-Aldrich) for 20 min. Foci were stained with a rabbit anti-spike monoclonal antibody (BS-R2B12, GenScript A02058) at 0.5 μg ml^−1^ in a permeabilization buffer containing 0.1% saponin (Sigma-Aldrich), 0.1% BSA (Sigma-Aldrich) and 0.05% Tween-20 (Sigma-Aldrich) in PBS. Plates were incubated with primary antibody overnight at 4 °C, and then washed with wash buffer containing 0.05% Tween-20 in PBS. Secondary goat anti-rabbit antibody conjugated to horseradish peroxidase (Abcam ab205718) was added at 1 μg ml^−1^ and incubated for 2 h at room temperature with shaking. TrueBlue peroxidase substrate (SeraCare 5510-0030) was then added at 50 μl per well and incubated for 20 min at room temperature. Plates were imaged in an ImmunoSpot Ultra-V S6-02-6140 Analyzer ELISPOT instrument with BioSpot Professional built-in image analysis (Cellular Technology Limited).

### Statistics and fitting

Statistical methods were not used to predetermine sample size, and blinding and randomization were not used. All statistics and fitting were performed using custom code in MATLAB v.2019b. Neutralization data were fitted to:

$${\rm{Tx}}=1/1+(D/{{\rm{ID}}}_{50}).$$

Here Tx is the number of foci normalized to the number of foci in the absence of plasma on the same plate at dilution *D*, and ID_50_ is the plasma dilution giving 50% neutralization. FRNT_50_ = 1/ID_50_. Values of FRNT_50_ < 1 are set to 1 (undiluted), the lowest measurable value. We note that the most concentrated plasma dilution was 1:25 and therefore FRNT_50_ < 25 data were extrapolated. To calculate CIs, FRNT_50_ or fold change in FRNT_50_ per participant was log transformed and the arithmetic mean plus 2 s.d. and the arithmetic mean minus 2 s.d. were calculated for the log-transformed values. These were exponentiated to obtain the upper and lower 95% CIs on the geometric mean FRNT_50_ or the fold change in FRNT_50_ geometric means.

### Reporting summary

Further information on research design is available in the Nature Research Reporting Summary linked to this paper.

## Online content

Any methods, additional references, Nature Research reporting summaries, source data, extended data, supplementary information, acknowledgements, peer review information; details of author contributions and competing interests; and statements of data and code availability are available at 10.1038/s41586-022-04830-x.

### Supplementary information

Reporting Summary

### Source data

Source Data Figs. 1–3

## Figures and Tables

**Extended Data Table 1:**
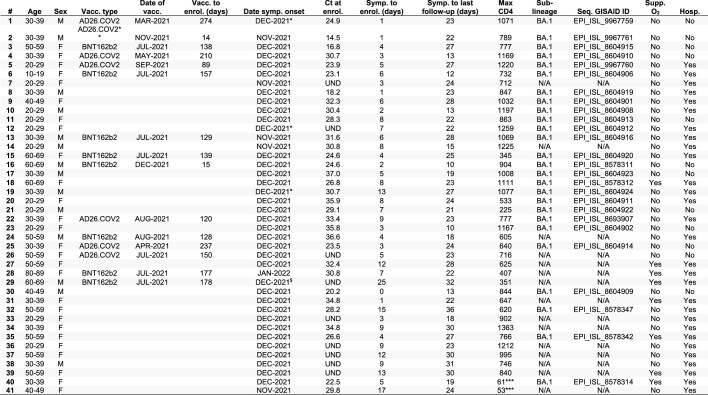
Characteristics of Omicron/BA.1 infected participants

Ct enrol.: qPCR cycle threshold for SARS-CoV-2 at enrollment. Symptoms to enrol.: time between symptoms onset and study enrolment. Symp. to last follow-up: time between symptoms onset and last follow-up visit. Max CD4: maximum CD4 count per microliter blood across all study visits. Supp O_2_: participant required supplemental oxygen during the study. Hosp.: participant hospitalized during the study. UND: Undetectable Ct. N/A: Not available; sequencing failed, usually due to insufficient virus substrate. *Reported previous infection. **Boosted with Ad26.CoV2.S in Nov-2021. ***Participants with persistent low CD4 count and uncontrolled HIV viremia indicative of advanced HIV disease and immune suppression. Excluded from analysis. ^$^Deceased.

**Extended Data Table 2:**
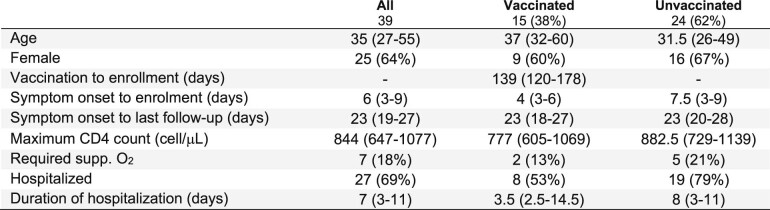
Summary characteristics of Omicron/BA.1 infected participants

Values are median (IQR). Hospital stay calculated to last inpatient study visit.

**Extended Data Table 3:**
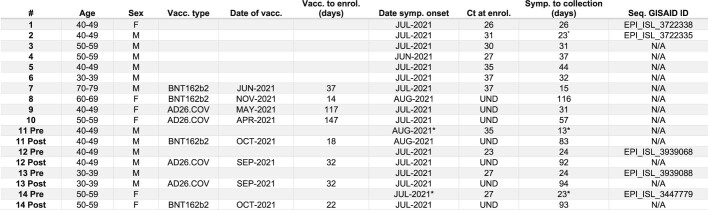
Characteristics of Delta infected participants

*Asymptomatic, date of diagnostic swab used instead of symptoms onset. Ct enrol: qPCR cycle threshold for SARS-CoV-2 at enrollment. UND: undetectable. Pre: sample taken pre-vaccination. Post: sample taken post-vaccination for participants with a pre-vaccination sample. N/A: not available.

## Data Availability

Sequences of outgrown Omicron sublineages have been deposited in GISAID (https://www.gisaid.org/) with accessions EPI_ISL_7886688 (Omicron BA.1), EPI_ISL_9082893 (Omicron BA.2) and EPI_ISL_602626.1 (ancestral/D614G). Delta (EPI_ISL_3118687) and Beta (EPI_ISL_678615) isolates have been described previously^15^. Raw images of the data are available upon reasonable request. Source data are provided with this paper.
