# Supplementary material for: Omicron infection enhances Delta antibody immunity in vaccinated persons
Source: Nature. 2022 May 6;607(7918):356–9. doi: 10.1038/s41586-022-04830-x (PMC9279144; doi:10.1038/s41586-022-04830-x)
Supplement: Supplementary file 1 — Reporting Summary [file 41586_2022_4830_MOESM1_ESM.pdf]

nature portfolio

Corresponding author(s): Alex Sigal

Last updated by author(s): Apr 18, 2022

# Reporting Summary

Nature Portfolio wishes to improve the reproducibility of the work that we publish. This form provides structure for consistency and transparency in reporting. For further information on Nature Portfolio policies, see our Editorial Policies and the Editorial Policy Checklist.

## Statistics

For all statistical analyses, confirm that the following items are present in the figure legend, table legend, main text, or Methods section.

| n/a | Confirmed | |
|---|---|---|
| ☐ | ☒ | The exact sample size (*n*) for each experimental group/condition, given as a discrete number and unit of measurement |
| ☐ | ☒ | A statement on whether measurements were taken from distinct samples or whether the same sample was measured repeatedly |
| ☐ | ☒ | The statistical test(s) used AND whether they are one- or two-sided<br>*Only common tests should be described solely by name; describe more complex techniques in the Methods section.* |
| ☒ | ☐ | A description of all covariates tested |
| ☒ | ☐ | A description of any assumptions or corrections, such as tests of normality and adjustment for multiple comparisons |
| ☐ | ☒ | A full description of the statistical parameters including central tendency (e.g. means) or other basic estimates (e.g. regression coefficient) AND variation (e.g. standard deviation) or associated estimates of uncertainty (e.g. confidence intervals) |
| ☐ | ☒ | For null hypothesis testing, the test statistic (e.g. *F*, *t*, *r*) with confidence intervals, effect sizes, degrees of freedom and *P* value noted<br>*Give P values as exact values whenever suitable.* |
| ☒ | ☐ | For Bayesian analysis, information on the choice of priors and Markov chain Monte Carlo settings |
| ☒ | ☐ | For hierarchical and complex designs, identification of the appropriate level for tests and full reporting of outcomes |
| ☒ | ☐ | Estimates of effect sizes (e.g. Cohen's *d*, Pearson's *r*), indicating how they were calculated |

*Our web collection on statistics for biologists contains articles on many of the points above.*

## Software and code

Policy information about availability of computer code

| | |
|---|---|
| Data collection | Participant data was collected using REDCap version 11.1.29 |
| Data analysis | All statistics and fitting were performed using MATLAB v.2019b |

For manuscripts utilizing custom algorithms or software that are central to the research but not yet described in published literature, software must be made available to editors and reviewers. We strongly encourage code deposition in a community repository (e.g. GitHub). See the Nature Portfolio guidelines for submitting code & software for further information.

## Data

Policy information about availability of data

All manuscripts must include a data availability statement. This statement should provide the following information, where applicable:

- Accession codes, unique identifiers, or web links for publicly available datasets
- A description of any restrictions on data availability
- For clinical datasets or third party data, please ensure that the statement adheres to our policy

Sequences of outgrown Omicron sub-lineages have been deposited in GISAID with accession EPIISL_7886688 (Omicron/BA.1) and EPIISL9082893 (Omicron/BA.2). Delta, Beta, and ancestral SARS-CoV-2 isolates have been previously described (15). Raw images of the data are available upon reasonable request.



March 2021

# Field-specific reporting

Please select the one below that is the best fit for your research. If you are not sure, read the appropriate sections before making your selection.

☒ Life sciences ☐ Behavioural & social sciences ☐ Ecological, evolutionary & environmental sciences

For a reference copy of the document with all sections, see nature.com/documents/nr-reporting-summary-flat.pdf

# Life sciences study design

All studies must disclose on these points even when the disclosure is negative.

| | |
|---|---|
| Sample size | Samples size was not predetermined. We offered study enrollment to all participants who met the inclusion/exclusion criteria |
| Data exclusions | We excluded two samples from participants who were immune suppressed due to advanced HIV disease based on a low CD4 count and uncontrolled HIV viremia. |
| Replication | Repeated in independent experiments on different days in sets of paired experiments to which always included Omicron/BA.1. Two experiments performed for all variants and 6 experiments performed for BA.1. Geometric mean of measurements per participant was used. |
| Randomization | No randomization. |
| Blinding | No blinding |

# Reporting for specific materials, systems and methods

We require information from authors about some types of materials, experimental systems and methods used in many studies. Here, indicate whether each material, system or method listed is relevant to your study. If you are not sure if a list item applies to your research, read the appropriate section before selecting a response.

## Materials & experimental systems

| n/a | Involved in the study | |
|---|---|---|
| ☐ | ☒ | Antibodies |
| ☐ | ☒ | Eukaryotic cell lines |
| ☒ | ☐ | Palaeontology and archaeology |
| ☒ | ☐ | Animals and other organisms |
| ☐ | ☒ | Human research participants |
| ☒ | ☐ | Clinical data |
| ☒ | ☐ | Dual use research of concern |

## Methods

| n/a | Involved in the study | |
|---|---|---|
| ☒ | ☐ | ChIP-seq |
| ☒ | ☐ | Flow cytometry |
| ☒ | ☐ | MRI-based neuroimaging |

## Antibodies

| | |
|---|---|
| Antibodies used | Foci were stained with a rabbit anti-spike monoclonal antibody (BS-R2B12, GenScript A02058) at 0.5 µg/mL. Secondary goat anti-rabbit horseradish peroxidase (Abcam ab205718) antibody was added at 1 µg/mL |
| Validation | Information sheet for A02058 at https://www.genscript.com/antibody/A02058-MonoRab_SARS_CoV_2_Spike_S1_Antibody_BS_R2B12_mAb_Rabbit.html. Information sheet for ab205718: https://www.abcam.com/goat-rabbit-igg-hl-hrp-ab205718.html |

## Eukaryotic cell lines

Policy information about cell lines

| | |
|---|---|
| Cell line source(s) | Vero E6 cells (ATCC CRL-1586) obtained from Cellonex in South Africa. The H1299-E3 (H1299-ACE2, clone E3) cell line was derived from H1299 as described in our previous work. H1299 was a gift from M. Oren, originally obtained from ATCC (CRL-5803) |
| Authentication | Cell lines have not been authenticated. |
| Mycoplasma contamination | The cell lines have been tested for mycoplasma contamination and are mycoplasma negative. |
| Commonly misidentified lines (See ICLAC register) | None |

## Human research participants

Policy information about studies involving human research participants

| | |
|---|---|
| Population characteristics | Participant characteristics are listed per participant in Table S1 and S3 and summarized in Table S2. |
| Recruitment | Blood samples were obtained from hospitalized adults with PCR-confirmed SARS-CoV-2 infection and/or vaccinated individuals who were enrolled in a prospective cohort study approved by the Biomedical Research Ethics Committee at the University of KwaZulu–Natal. Investigators were blinded to participant information. |
| Ethics oversight | Study approved by the Biomedical Research Ethics Committee at the University of KwaZulu–Natal (reference BREC/00001275/2020). Use of residual Omicron/BA.1 swab sample was approved by the University of the Witwatersrand Human Research Ethics Committee (HREC) (ref. M210752). Use of swab sample to isolate Omicron/BA.2 was collected as part of the "COVID-19 transmission and natural history in KwaZulu-Natal, South Africa: Epidemiological Investigation to Guide Prevention and Clinical Care" Centre for the AIDS Programme of Research in South Africa (CAPRISA) study and approved by the Biomedical Research Ethics Committee at the University of KwaZulu–Natal (reference 201 BREC/00001195/2020, BREC/00003106/2021). |

Note that full information on the approval of the study protocol must also be provided in the manuscript.
